# 2D/2D Heterojunctions for Catalysis

**DOI:** 10.1002/advs.201801702

**Published:** 2019-01-30

**Authors:** Juan Su, Guo‐Dong Li, Xin‐Hao Li, Jie‐Sheng Chen

**Affiliations:** ^1^ School of Chemistry and Chemical Engineering Shanghai Jiao Tong University Shanghai 200240 P. R. China; ^2^ State Key Laboratory of Inorganic Synthesis and Preparative Chemistry College of Chemistry Jilin University Changchun 130012 P. R. China

**Keywords:** 2D nanojunctions, electrocatalysis, organic synthesis, photocatalysis, synthesis

## Abstract

2D layered materials with atomic thickness have attracted extensive research interest due to their unique physicochemical and electronic properties, which are usually very different from those of their bulk counterparts. Heterojunctions or heterostructures based on ultrathin 2D materials have attracted increasing attention due to the integrated merits of 2D ultrathin components and the heterojunction effect on the separation and transfer of charges, resulting in important potential values for catalytic applications. Furthermore, 2D/2D heterostructures with face‐to‐face contact are believed to be a preferable dimensionality design due to their large interface area, which would contribute to enhanced heterojunction effect. Here, the cutting‐edge research progress in 2D/2D heterojunctions and heterostructures is highlighted with a specific emphasis on synthetic strategies, reaction mechanism, and applications in catalysis (photocatalysis, electrocatalysis, and organic synthesis). Finally, the key issues and development perspectives in the applications of 2D/2D layered heterojunctions and heterostructures in catalysis are also discussed.

## 1 Introduction

The definition of heterojunction, originally developed from the semiconductor–semiconductor (S–S) junction, has now been extended to the scope of metal–semiconductor (M–S) junction and even untypical heterostructures of semiconductors and ionic conductors.^1^, ^2^ Generally speaking, the coupled interface of two components in a heterojunction could generate a band alignment or a rectifying contact after the equilibration of the Fermi levels (or work functions) at the interface according to the Anderson's rule or Schottky–Mott rule for S–S or M–S junctions, respectively.^1^, ^2^, ^3^ There is a consensus in the literature that the relocalization of charge carriers at the interface of heterojunctions may facilitate the catalytic performance of the as‐fabricated materials or devices.^1^, ^2^, ^4^, ^5^, ^6^, ^7^, ^8^, ^9^, ^10^, ^11^, ^12^, ^13^, ^14^, ^15^, ^16^, ^17^, ^18^

**Figure 1:**
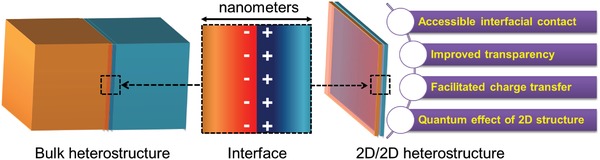
Schematic structures of bulk heterojunction and 2D/2D heterojunction with coupled interface.

Dimensionality, as one of the structural parameters, also determines properties of heterostructure to a large extent.^19^, ^20^ The scale of the space charge region at the interface usually lies between several and a few tens of nanometers (**Figure** **1** middle). It should be noted that the interfacial effect of bulk heterostructures/heterojunctions with the interfacial part buried inside the bulk (Figure 1 left) on the redox ability of the hybrids is negligible. The thin structure of 2D materials might be an ideal flat to make full use of the electron relocalization at the interfacial part of a heterojunction to modify its redox ability for possible applications in catalysis (Figure 1 right).^15^, ^21^, ^22^

Since the first synthesis of graphene (GR) with astonishing properties in 2004,^20^ “2D materials,” especially the atomically layered ones, as a new class of materials attracted great interest among researchers and simultaneously enormous efforts for the study and development of alternative layered materials. It has been demonstrated that ultrathin 2D materials possess unique physical, chemical, and electric properties, which are usually very different from those of their bulk counterparts,^4^, ^23^, ^24^, ^25^, ^26^, ^27^, ^28^ e.g., large specific surface area, excellent bendability, unique optical bandgap structures, strong light–matter interactions, and high carrier mobility. Furthermore, most 2D nanosheets are easily fabricated into flexible films with uniform thickness, which are programmable platforms for practical applications. Although heterojunction based on various dimensions (including 2D/2D, 2D/3D, and 3D/3D) with exposed interface of rectifying contact are all possible effective catalysts, 2D/2D heterojunctions possess unique advantages for catalysis (Figure 1):^4^, ^5^, ^6^, ^7^, ^8^, ^9^, ^11^, ^12^, ^13^ i) rich catalytic active sites owing to both large surface/interface area and ultrathin thickness of 2D components; ii) charge transfer would be facilitated due to the low intrinsic resistance and short transport path at one dimension of ultrathin 2D components; and iii) transparency result from ultrathin thickness is beneficial for light absorption.

**Figure 2:**
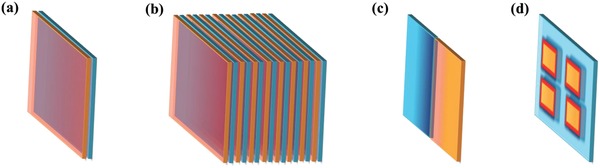
a–d) Schematic illustration of typical 2D/2D heterostructures with different contact interfaces.

As a result, the design and utilization of 2D/2D layered heterostructures has promptly become one of the hottest research topics. In recent years, the family of 2D ultrathin layered materials has grown appreciably. Beyond the currently used 2D ultrathin nanosheets, such as graphene, transition metal dichalcogenides, and noble metals,^2^, ^7^, ^23^, ^24^, ^29^ there are many other ultrathin 2D nanosheets with varying physical and electronic properties developed in the past few years, e.g., metal oxides,^30^ hexagonal boron nitride (h‐BN),^21^, ^31^ black phosphorus,^32^ metal‐organic frameworks (MOFs),^33^ covalent‐organic frameworks (COFs),^34^ and organic crystals.^35^ The above‐mentioned achievements provide a wide variety of building blocks and synthetic techniques for the fabrication of 2D/2D heterostructures. Furthermore, 2D/2D heterostructures also can be designed with various contact interfaces in vertical or lateral directions as shown in **Figure** **2**.^4^, ^5^, ^6^, ^7^, ^8^, ^9^, ^11^, ^12^, ^13^, ^17^ In vertical direction, 2D/2D heterostructures with face‐to‐face interface contact can be constructed by stacking two (Figure 2a) or multiple (Figure 2b) monolayer flakes of different materials. The heterostructures can be gradually engineered with atomic precision by adjusting the relative orientation between the individual elements.^7^, ^13^, ^17^, ^36^ In lateral direction, 2D/2D heterostructures with cross‐section interface contact can be constructed through epitaxial growth methods.^13^, ^15^, ^37^, ^38^ Both paralleled and patterned contacts can be realized like the heterostructures in Figure 2c,d. Different types of contact interface can be designed for different heterojunction effect on catalytic active sites and performance.

A number of excellent reviews on 2D layered materials focusing on synthesis, physicochemical properties, and applications have been published in the past few years.^23^, ^24^, ^25^, ^26^, ^27^, ^39^ There are also some comprehensive reviews on the heterostructures based on various 2D materials.^4^, ^5^, ^6^, ^7^, ^8^, ^9^, ^11^, ^12^, ^13^ The advantages and potential applications of 2D/2D heterostructures have also attracted increasing recognition and awareness. A comprehensive review focusing on the fabrication of 2D/2D heterojunctions for catalytic applications is thus necessary to provide researchers with a better understanding on basic conception and most recent progress. Here, we present a comprehensive overview of the recent progress in design of 2D/2D layered heterostructures for on‐demand modification of the catalytic activity with the assistance of interfacial contact. The catalytic applications reviewed in this work involve photocatalysis, electrocatalysis, and organic synthesis. Finally, the key issues and development prospects of 2D/2D heterostructures for their catalytic applications are also discussed.

## 2 Synthetic Methods

Since 2D materials successfully synthesized by mechanical exfoliation, an increasing number of synthetic methods, like chemical exfoliation and chemical vapor deposition (CVD) methods, have been developed to obtain 2D materials with various nanostructures. For example, there are more opportunities to obtain 2D materials with high crystallinity but small‐scale production through mechanical exfoliation method; chemical exfoliation methods such as electrochemical^40^ and solvents assistance^28^, ^41^ exfoliation are apt to realize large‐scale production of 2D materials but without precise control of lateral size and thickness; and chemical vapor deposition method can form monolayer 2D materials^42^ and is also suitable for their wafer‐scale synthesis.^43^ Thanks to the synthetic variety of 2D materials, a wide variety of building blocks and techniques are applied to fabricate 2D/2D heterostructures. Here, the synthesis schemes of 2D/2D heterostructures are categorized as “ex situ assembly methods” and “in situ growth methods,” which are introduced in detail as following.

### 2.1 Ex Situ Assembly Methods

The “ex situ assembly methods” are mainly applied to engineer 2D/2D layered heterostructures based on presynthesized 2D layered materials, which are mechanically/chemically exfoliated from bulk materials or isolated from the synthetic/grown 2D layers, and then manually stacked to form 2D/2D heterostructures, whose 2D ultrathin components are combined together based on weak van der Waals (vdW) interlayer force.^44^, ^45^, ^46^, ^47^, ^48^, ^49^, ^50^

**Figure 3:**
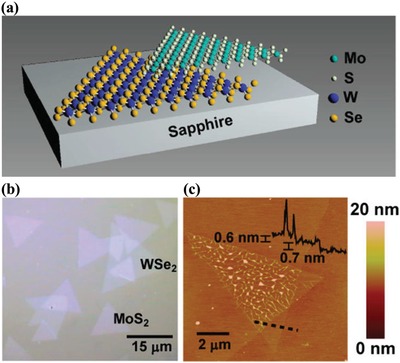
a) Schematic illustration of the components of MoS_2_/WSe_2_ heterostructures (MoS_2_ monolayers are stacked onto WSe_2_ monolayers); b) optical micrograph of MoS_2_/WSe_2_ heterostructures after thermal treatment; and c) AFM image of the MoS_2_/WSe_2_ heterostructures (the thickness of MoS_2_ or WSe_2_ monolayers is 0.6–0.7 nm according to the cross‐sectional height profile). Reproduced with permission.^49^ Copyright 2014, American Chemical Society.

Chiu et al. successfully fabricated a vertical 2D/2D MoS_2_/WSe_2_ heterostructure through stacking MoS_2_ and WSe_2_ triangular layers and subsequent thermal treatment.^49^ Specifically, single crystalline WSe_2_ and MoS_2_ monolayers with few tens of micrometers are first prepared by CVD on c‐plane sapphire substrates.^51^ Polymeric Methyl Methacrylate (PMMA) was first used to detach as‐synthesized MoS_2_; PMMA layer was spin‐coated on MoS_2_/substrate, and then dipped in NaOH together to be released from the substrates; and PMMA was finally removed after the PMMA supported‐MoS_2_ was transferred onto WSe_2_ flakes (**Figure** **3**a). The stacked layers of mechanically stacked MoS_2_/WSe_2_ can be distinguished from the optical micrograph in Figure 3b. The results of atomic force microscope (AFM) measurement reveal that WSe_2_ and MoS_2_ components are monolayers with a thickness of 0.6–0.7 nm and without cracks or defects even after thermal treatment in the presence of H_2_/Ar gas (Figure 3c).

In another work, Zhang et al. developed a MoTe_2_/MoS_2_ atomically thin 2D/2D heterostructure by transfer technique without any subsequent thermal treatment process to strength the interaction of the two components.^44^ MoS_2_ monolayers as the bottom layer are first synthesized by chemical vapor deposition, and MoTe_2_ monolayer crystals are mechanically exfoliated by repeatedly peeling. MoS_2_ and MoTe_2_ monolayers are affixed together by poly(methyl methacrylate)/poly(vinyl acetate) (PMMA/PVA) double layers, which are subsequently removed by acetone washing. MoTe_2_ and MoS_2_ 2D ultrathin crystals are respectively stabilized by strong covalent bonds, while they are stacked together by relatively weak van‐der‐Waals‐like forces. The interlayer infrared excitation first demonstrated in the as‐prepared MoTe_2_/MoS_2_ monolayer heterostructures should be attributed to the type‐II heterojunction induced by the interlayer interactions between MoTe_2_ and MoS_2_ monolayer.

**Figure 4:**
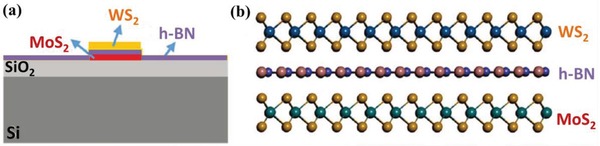
a) Schematic diagram and b) atomic model of WS_2_/MoS_2_ heterostructures, h‐BN layers are introduced to control the separation distance between WS_2_ and MoS_2_ layers. Reproduced with permission.^45^ Copyright 2018, WILEY‐VCH.

Besides the selection and coordination of building blocks, the interlayered separation distance and the stacking lattice orientation of 2D/2D layered heterostructures are critical for their properties. Xu et al. fabricated WS_2_/MoS_2_ layered heterostructures by ex situ assembling resynthesized WS_2_ and MoS_2_ monolayer materials.^45^ The interlayer interactions of WS_2_ and MoS_2_ monolayers are studied by photoluminescence (PL) spectroscopy, resulting that both the input excitation power and the separation distance play the key role. In this work, insulating h‐BN layers were produced by CVD and introduced between WS_2_ and MoS_2_ layers in heterostructures to control the separation distance between WS_2_ and MoS_2_ layers (**Figure** **4**). Through adjusting the layer number of h‐BN, the interlayer interactions are successfully tuned in fine‐scale in the as‐fabricated heterostructures. In addition, the stacking lattice orientation may also obviously influence the interface contact and thus determine the properties of heterostructures.^46^, ^47^, ^48^, ^49^ Dean et al. demonstrated that h‐BN layers with atomic flat and nearly free charge trapping showed more advantages to be used as substrate for graphene compared with amorphous SiO_2_ substrates.^46^ Graphene/h‐BN layered heterostructures show obviously higher carrier mobility due to their less doping, reduced roughness, and improved chemical stability compared with graphene/SiO_2_.

### 2.2 In Situ Growth Methods

“In situ growth methods” are the direct growth of 2D/2D layered composites or the formation of one 2D layered materials on the surface of another ones through one‐step growth or multi‐step conversion approaches, such as the techniques of CVD,^7^, ^52^, ^53^ wet‐chemical synthesis,^17^, ^54^ and nanoconfinement methods.^21^ Various synthetic methods may contribute to different heterojunction interface and application potentials based on specific principles.

Though “ex situ assembly methods” produce many 2D/2D layered heterostructures suitable for research and some applications, there are still disadvantages undesired for some important applications, including unavoidable interface contaminants, random relative alignment, and weak interfacial interaction. “In situ growth methods” are alternative techniques for the construction of promising 2D/2D layered heterostructures.^6^, ^7^ There are normally two ways for the in situ growth of 2D/2D layered heterostructures: i) epitaxial growth of 2D crystals on top or bare region of mechanically transferred or grown 2D layered materials; and ii) direct growth of heterostructures.

**Figure 5:**
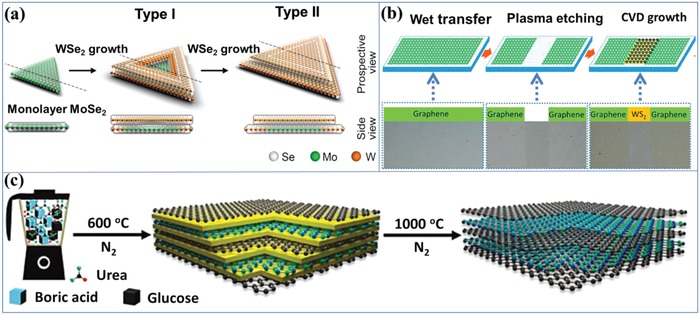
a) Schematic illustration of the MoSe_2_‐templated WSe_2_ growth. Reproduced with permission.^55^ Copyright 2015, American Chemical Society. b) Synthetic paths for preparing graphene/WS_2_ layered heterostructures (dashed boxes: the corresponding optical images of the samples). Reproduced with permission.^53^ Copyright 2017, American Chemical Society. c) Synthetic paths for preparing h‐BN/G layered heterostructures. Reproduced with permission.^21^ Copyright 2018, WILEY‐VCH.

Based on one kind of 2D layered materials, epitaxial growth of both lateral and vertical 2D/2D heterostructures can be realized by CVD techniques.^7^, ^13^, ^53^, ^55^ In typical CVD, the substrate is exposed to volatile precursors, which would react or decompose on the surface of substrate. In this process, the volatile by‐products are removed by the gas flow in the reaction system. CVD is believed to be a promising technique for synthesizing 2D atomically thin heterostructures due to the fine tuning of composition, thickness, and interface. Gong et al. developed a two‐step CVD method for growing WSe_2_/MoSe_2_ vertical layered heterostructures where WSe_2_ was epitaxially grown on the edge and top surface of presynthesized MoSe_2_ by CVD.^55^ As shown in **Figure** **5**a, WSe_2_ would grow from the edge regions of monolayer MoSe_2_, forming WSe_2_ bilayer and WSe_2_/MoSe_2_ bilayer heterostructures (Figure 5a Type I). Further extending the growth time, MoSe_2_ was eventually covered with WSe_2_, forming WSe_2_/MoSe_2_ heterostructures with additional WSe_2_ monolayer and bilayer at the edges of heterostructure (Figure 5a Type II). The as‐obtained heterostructures' size is as large as 169 µm; furthermore, the cross‐contamination is effectively minimized. In another example, Zheng et al. developed lateral graphene−WS_2_ layered heterostructures with large area through a facial CVD technique.^53^ As shown in Figure 5b, large‐area CVD‐grown graphene as template was transferred onto a sapphire substrate and patterned using conventional photolithography and etched by oxygen plasma. Subsequently, monolayer WS_2_ selectively grows on the bare sapphire among the etched region of graphene to form continuous monolayer films with graphene/WS_2_ heterostructures.

Without 2D layer as template, 2D/2D heterostructures also can be obtained by direct synthetic method.^13^, ^17^, ^21^, ^56^ Recently, Zhang et al. developed a modified nanoconfinement method for the direct synthesis of 2D/2D layered heterostructures composed of graphene and h‐BN layers (h‐BN/G).^21^ In a typical synthesis (Figure 5c), the mixture of glucose (carbon source), boric acid (boron source), and urea (leaving reagent) was used as starting materials, which generated the key precursor with C‐rich domains and B‐rich domains. After the high temperature treatments at 1000 °C under nitrogen atmosphere, h‐BN/G layered heterostructures are directly synthesized. During the synthetic process, a yellow intermediate graphitic carbon nitride (g‐C_3_N_4_) exists as lamellar template for the further growth of graphene and h‐BN in the temperature range of 600–750 °C. In another example, the direct growth of graphene/Mo_2_C vertical layered heterostructures also can be realized by CVD techniques.^56^ The as‐obtained 2D/2D layered heterostructures are quite high‐quality due to their uniformly well‐aligned lattice orientation and strong interface coupling.

## 3 Roles of the Interfacial Electron Relocalization in Modifying the Catalytic Activity of 2D/2D Heterojunctions

Besides the influence of compositions and dimensionality of constituent 2D materials, the highly coupled interface, especially based on ultrathin 2D materials, always plays key role in facilitating the catalytic performance of 2D/2D heterostructures via electron relocalization induced i) ultrafine nanostructure or interfacial defects and thus novel active sites; ii) band bending and thus enhanced redox power of active sites; iii) high‐speed electron transfer path at interface.

### 3.1 Typical Electronic Structures of Heterojunctions

Heterojunctions with various geometrical and energy band alignments would exhibit electronic properties or functionalities which are macroscopically different from their constituent materials.^1^, ^2^, ^8^ Based on 2D layered materials, heterojunctions are engineered by constructing coupled contact at interface, which would influence the behavior or distribution of charges and thus modify the physicochemical properties and functions.^5^, ^6^, ^7^, ^8^, ^9^, ^10^, ^11^, ^12^, ^13^, ^18^ The electronic band structures of typical S–S junctions and M–S junctions were depicted in Figure 5.

**Figure 6:**
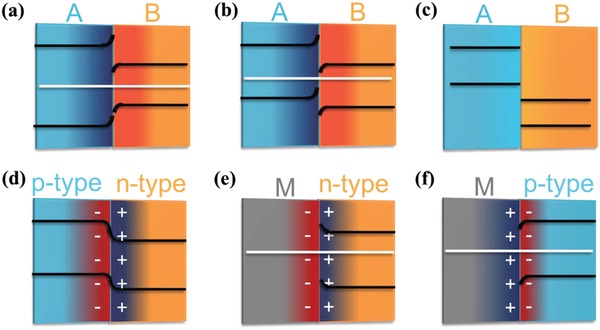
a–f) Schematic illustration of electronic band structures of typical S–S junctions and M–S junctions.

There are generally three types of band alignments at the interface of S–S heterojunctions (**Figure** **6**a–c): type‐I heterojunctions with a straddling gap (Figure 6a), type‐II heterojunctions with a staggered gap (Figure 6b), and type‐III heterojunctions with a broken gap (Figure 6c).^1^, ^57^ In type‐I heterojunctions, the conduction band (CB) and the valence band (VB) levels of semiconductor B are between those of semiconductor A. The electrons and holes are apt to transfer to and accumulate at the CB and the VB levels of semiconductor B, respectively, among the interface of type‐I heterojunctions. In type‐II heterojunctions, both the CB and the VB levels of semiconductor A are higher than the corresponding levels of semiconductor A, respectively. The electrons are apt to transfer to semiconductor B, while the holes are apt to accumulate at semiconductor A among the interface of type‐II heterojunctions. In type‐III heterojunctions, both the CB and VB levels of semiconductor A are higher than those of semiconductor A without the formation of space charge zone for further modification of redox power. The band bending at the interface of type‐I and type‐II heterojunctions even at zero bias makes it possible to tailor the redox power of semiconductor A or B for specific purpose.

When different dopants are introduced into a semiconductor and even different semiconductors, it is also possible to construct a p–n junction (Figure 6d), resulting in an interfacial potential difference across the grain boundary even without an external bias. Moreover, p‐type and n‐type semiconductors could also be applied to construct rectifying junctions with metals or semimetals. Semiconductor and metal are always not independent system, especially based on their appropriate band structures and work functions. The supported metal can exhibit highly enhanced chemical reactivity due to the electronic interaction and electron transfer induced by Schottky effect at the planar metal–semiconductor interfaces. Under the effect of Schottky junction, electrons will flow until the Fermi level on both sides of the interface is the same (Figure 6e,f).^8^ Currently, increasing efforts have been focused on engineering heterostructures between metal and semiconductor, which not only proved to be a critical component in electronic and optoelectronic, but also exhibited highly enhanced catalytic activity.^8^, ^58^ The M–S heterostructures with tailorable catalytic activity for specific reaction via the controllable Schottky effect was nominated as Mott–Schottky catalyst by Li and Antonietti.^8^, ^58^ In heterogeneous catalysis, the semiconductor in metal–semiconductor Mott–Schottky catalyst plays the same role as ligands in homogeneous catalysis. A change of electron density at active metal centers and band bending of the semiconductor side induced by Schottky junctions would dominate their final catalytic activity. Similarly, 2D/2D S–S heterojunction could also be applied as possible catalysts even though most S–S heterojunctions were applied in various electronic devices at the moment.^4^, ^5^, ^6^, ^7^

### 3.2 Modification of Active Sites via Interfacial Effect of 2D/2D Heterojunctions

#### 3.2.1 Structure Engineering Induced Novel Active Sites

In the research field of functional materials, engineering the structures, including size, surface area, porosity, crystal facet, and defects, has always been an effective strategy to enhancing catalysts' performance and generating novel active sites. The interfacial effect of 2D/2D heterostructures has been applied to engineer the mesoscale structure of 2D/2D heterojunctions to introduce a rich amount of accessible active sites for specific catalytic reactions.

**Figure 7:**
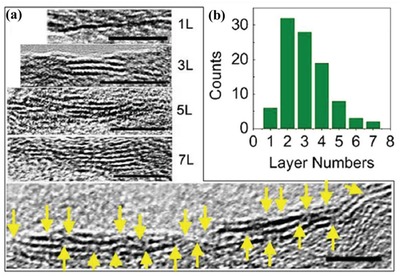
a) HRTEM images of MoS_2_‐CP heterostructures with different layer numbers (yellow arrows: defects; scale bar: 5 nm); b) The distribution of MoS_2_ with different layer number in MoS_2_‐CPs. Reproduced with permission.^16^ Copyright 2015, Elsevier.

Ye et al. successfully construct a highly efficient hydrogen evolution reaction (HER) electrode materials with heterostructure (MoS_2_‐CPs) composed of tiny MoS_2_ nanosheets and hybrid carbon papers (CPs).^16^ Layered MoS_2_, as a cheap alternative of platinum with noble‐metal‐like catalytic activities, exhibits significantly higher electrocatalytic performance for HER compared with that of bulk phase, because the catalytic active sites mainly lie at the edges of 2D layers. In this work, the small size (<15 nm) and few‐layer thickness (2–3 layers) of the as‐formed patched MoS_2_ nanosheets ensure the abundance of exposed edges and defects as electrocatalytic active sites (**Figure** **7**a,b) for efficient electrocatalytic activity. Furthermore, patched few‐layer MoS_2_ catalysts with rather small size are engineered and uniformly distributed on CPs. As a control experiment, the aggregates of MoS_2_ with large size and thick thickness are formed without CPs as supports. Therefore, both the homogeneous distribution and special structure engineering (small, few‐layer, and defective) of MoS_2_ nanosheets, which are beneficial for catalytic performance, should be attributed to presence of CP supports and thus resulting in interfacial effect of highly coupled MoS_2_ nanosheets and CPs.

#### 3.2.2 Enhanced Redox Power of Active Sites

The rectifying contact at the interface of a heterojunction had very important role in modifying the redox ability of semiconductor components with already known active sites due to the formation of static electronic field and bended conduction/valance bands, even though the amounts of active sites were not changed too much.

**Figure 8:**
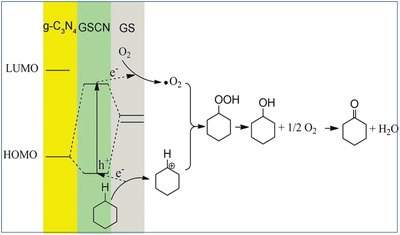
Schematic illustration of the oxidation mechanism of GSCN heterostructures. Reproduced with permission.^10^ Copyright 2011, American Chemical Society.

Metal‐free catalysts, owing to the advantages of low cost, high abundance, and excellent stability, attract increasing research interests. Although a series of metal‐free catalysts as green catalysts have been developed, the catalytic efficiency is still incomparable to that of metal‐based catalyst and limits their practical applications. Li et al. developed a series of metal‐free composite catalysts (GSCN) composed of graphene sheet (GS) and g‐C_3_N_4_ for the selective oxidation of sp^3^ C—H bonds to corresponding ketones through activating molecular oxygen.^10^ Compared with bare g‐C_3_N_4_ or GS alone, GSCN heterostructures exhibit significantly enhanced catalytic activity for the activation of molecular oxygen, which is reduced to form ^•^O_2_^−^ by the delocalized electrons from the lowest unoccupied molecular orbital (LUMO) of the C_3_N_4_. As shown in **Figure** **8**, the lowered highest unoccupied molecular orbital (HOMO) of C_3_N_4_ after the introduction of GS boosts the activation of the substrates. The charge‐transfer interactions at heterojunction interface can be confirmed by the significant decrease in photoluminescence intensity as compared with that of g‐C_3_N_4_. Most importantly, such an M–S dyad is a mild catalyst to boost the selective oxidation of cyclohexane into ketones without formation of overoxidation products.

In another work, an atomic‐scale heterostructure (h‐BN‐C/G) composed of a boron nitride monolayer and graphene is developed and successfully promoted the activation of molecular oxygen for oxidative coupling of amines to imines.^21^ It was demonstrated that the redox power of actives sites was enhanced by the promoted separation of the photogenerated charge carriers for directly activating the molecular oxygen and substrate due to the construction of heterojunction interface in h‐BN‐C/G heterostructures. The interfacial electron redistribution of h‐BN‐C/G was confirmed by the gradually decreased photoluminescence intensity with increasing the proportion of graphene. A special reversible transfer behavior of the photogenerated charge carriers at the interface of h‐BN‐C/G was observed by using the transient photovoltage technique.

### 3.3 High‐Speed Electron Transfer Path at the 2D/2D Interface

Besides the direct influence of structure or defects on catalytic active sites, the transfer and separation of charges in catalysts play quite important role in the efficiencies of photo/electrocatalysis or photo‐electrocatalysis. Constructing 2D/2D heterojunction interface with strong interaction has been proved to be a feasible way to facilitate the high‐speed electron transfer in catalyst system, so as to enhance their catalytic performance.

Recently, Zhang et al. developed a highly coupled 2D/2D heterostructures (BV/CN) composed of few‐layer g‐C_3_N_4_ and monoclinic BiVO_4_ for the efficient photo‐electrochemical oxygen evolution reaction (OER).^15^ Monoclinic BiVO_4_ with a moderate bandgap and a positive enough valence has been proved to be an efficient catalyst for photocatalytic oxidation reactions, such as decomposition of organics in waste water and water splitting for O_2_ production, under visible‐light irradiation. However, its potential application in photo‐electrochemical water oxidation is limited by low electron–hole separation efficiency.^22^, ^59^ In this work, flexible g‐C_3_N_4_ nanolayers are highly coupled with BiVO_4_ nanolayers to act as a “pump” to extract the photogenerated electrons from BiVO_4_, which effectively promotes the incident photon‐to‐current conversion efficiency and the electron–hole separation under visible light irradiation. The choice of few‐layer g‐C_3_N_4_ in this work is quite critical, owing to that the ultrathin thickness comparable to the exciton Bohr radius at one dimension (4–8 nm) lifts the restriction of ultrahigh intrinsic resistance of g‐C_3_N_4_, and thus contributes to the successful extraction of electrons from BiVO_4_.

## 4 Applications in Catalysis

Although the materials in their 3D forms have long been used as catalysts, the materials with 2D layered structure exhibit considerable changes in their electronic structure and offer new opportunities for chemical/structural modification and catalytic reactions.^4^, ^23^, ^24^, ^25^, ^26^, ^27^ Design of heterostructures based on 2D layered materials is one of the promising routes for flexibly controlling materials' chemical reactivity, which would enhance catalytic performance and provide new possibilities in catalysis through the heterojunction effect on the transfer and separation of charge at interface.^4^, ^5^, ^6^, ^7^, ^8^, ^9^, ^10^, ^11^, ^12^, ^13^, ^29^, ^60^, ^61^, ^62^ Over the past decade, 2D/2D layered heterostructures have been investigated and applied in a variety of catalytic reactions, such as photocatalysis, electrocatalysis, and organic synthesis.

### 4.1 Photocatalysis

Facing the energy shortage and environmental pollution of human society, semiconductor‐based photocatalysis has great potential to assure the long‐term and sustainable development, due to the direct utilization of green solar energy both for producing valuable chemical fuels and degrading organic pollutants.^1^, ^2^, ^11^, ^12^, ^23^, ^29^, ^62^

Normally, there are four steps in a photocatalytic process: i) light absorption; ii) formation of photogenerated electron–hole pairs (e^−^–h^+^); iii) migration and recombination of photogenerated e^−^–h^+^; and iv) redox reactions at the surface of photocatalysts. Among them, the recombination of e^−^–h^+^ would create useless heat, which plays negative role in photocatalytic performance. Developing properly engineered 2D/2D heterostructures has been proved to be one of the most promising ways for enhancing photocatalytic activity, due to that the heterojunction interfacial effect can promote the separation and thus prolong the lifetime of photogenerated e^−^–h^+^ in catalyst, which directly or indirectly participates in the redox reaction in photocatalytic H_2_ production or organics degradation. Numerous efforts have been devoted to tune the 2D component or strengthen the interfacial acting force to develop highly efficient 2D/2D photocatalysts.^23^, ^29^, ^62^

#### 4.1.1 Photocatalytic H_2_ Production

Since in 1972 Fujishima and Honda discovered that the water splitting took place on a TiO_2_ electrode under ultraviolet (UV) light irradiation,^63^ simultaneously enormous efforts were devoted to photocatalytic H_2_ production. Photocatalytic H_2_ production is a process that produces H_2_ (and O_2_) through reducing or oxidizing the adsorbed water molecules by photogenerated electrons and holes at the surface of semiconductor catalysts. Due to the rapid recombination of the photogenerated e^−^–h^+^ in catalysts, the developed and reported hydrogen production efficiency was still far from the requirement of practical applications. 2D/2D layered composite photocatalysts with properly engineered heterojunctions are quite promising for enhancing hydrogen production efficiency through promoting the separation of photogenerated e^−^–h^+^.^64^, ^65^, ^66^, ^67^, ^68^, ^69^, ^70^, ^71^, ^72^

Compared with UV light with a small proportion of solar radiation, visible light (about 50% of solar radiation) driven photocatalysts are more promising for highly efficient sunlight utilization and photocatalytic activity. Zhang et al. developed a kind of “sheet‐on‐sheet” hierarchical heterostructures for visible‐light‐driven photocatalytic H_2_ production through in situ growth of ZnIn_2_S_4_ ultrathin nanosheets on sheet‐like g‐C_3_N_4_ surfaces.^64^ Although g‐C_3_N_4_ is one of the most promising photocatalysts due to its high stability, nontoxic nature, unique electronic structure, and low cost, its photocatalytic activity is limited by poor light‐harvesting efficiency and fast recombination of photogenerated charge carriers.

Through coupling visible‐active ZnIn_2_S_4_ ultrathin nanosheets, the above drawbacks are overcome based on heterojunction contact interface, which can induce efficient interfacial transfer of photogenerated e^−^–h^+^ from g‐C_3_N_4_ to ZnIn_2_S_4_ and hinder charge recombination based on the measurement results of surface photovoltage and photoluminescence of ZnIn_2_S_4_/g‐C_3_N_4_ heterostructures. Both the suppressed charge recombination on g‐C_3_N_4_ nanosheets and the increased photogenerated charge carriers in ZnIn_2_S_4_ nanosheets contribute to the remarkable enhancement on the photocatalytic activity of ZnIn_2_S_4_/g‐C_3_N_4_ heterostructures for H_2_ production. The enhanced photocatalytic performance should also be attributed to the increased surface active sites and extended light absorption through the combination of ZnIn_2_S_4_ nanosheets.

Overall water‐splitting photocatalysts also can be developed for the production of H_2_ and O_2_ simultaneously, but it is still a big challenge.^65^, ^69^ To satisfy the redox potential for overall water splitting, the photocatalysts' conduction band minimum should be more negative than the reduction potential of H^+^/H_2_ (0 V vs normal hydrogen electrode (NHE)) and the valence band maximum should be more positive than the oxidation potential of O_2_/H_2_O (1.23 V). Because of the overpotential associated with the charge transfer and gas evolution, the minimum band gap of photocatalysts for effective water splitting is always larger than theoretical value (1.23 eV). Liao et al. design and predict MoS_2_/AlN(GaN) ultrathin layered heterostructures as highly efficient visible‐light photocatalysts for overall water splitting.^65^ Hydrogen and oxygen can be produced separately at opposite surfaces of the heterostructures, because AlN(GaN) and MoS_2_ monolayers act as electron donor and electron acceptor in this heterojunction photocatalysts, respectively. Pristine MoS_2_ with a direct band gap of 1.9 eV is a promising visible‐light‐driven photocatalyst, and is proved to be inefficient for water splitting. The group III nitrides (AlN or GaN) monolayers with good chemical/thermal stability and high thermal conductivity are demonstrated to be a good choice to construct heterostructures based on MoS_2_ monolayers so as to enhance photocatalytic activity. In addition, there is only around 2% lattice mismatch among the hexagonal crystal structure of AlN(GaN) and MoS_2_ monolayers, which is a big advantage for the construction of heterostructures. The MoS_2_/AlN and MoS_2_/GaN heterostructures are predicted to be efficient photocatalysts under visible‐light irradiation due to the proper band gaps for water splitting and good optical absorption.

**Figure 9:**
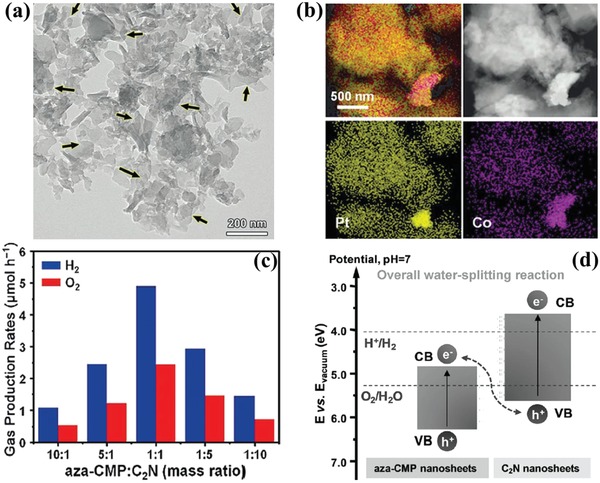
a) TEM image (arrows: overlapped interfaces), b) HAADF‐STEM images and elemental mapping (Pt‐labeled C_2_N and Co‐labeled aza‐CMP), c) overall water‐splitting performance, and d) schematic illustration of the electronic band structures of aza‐CMP/C_2_N heterostructures. Reproduced with permission.^69^ Copyright 2018, WILEY‐VCH.

Recently, polymer is believed to be one of the promising alternatives for photocatalytic overall water splitting due to its tunable molecular structures.^73^ To remedy the limitation of single‐component polymer with insufficient redox potentials,^74^ 2D/2D polymer heterojunction photocatalysts with Z‐scheme structure are designed through mimicking the two‐step excitation process of natural plants, in which the light‐driven two half reactions of water splitting and glucose synthesis are spatially separated.^75^ Polymer‐based Z‐scheme systems based on 2D materials have been proved to be an effective route to obtain proper energy levels for sufficient reaction potentials and enable efficient charge transfer in overall water splitting.^69^, ^76^ Wang et al. developed ultrathin heterostructures (aza‐CMP/C_2_N) composed of aza‐conjugated microporous polymers (CMP) and C_2_N nanosheets as 2D polymer‐based Z‐scheme systems for efficient photocatalytic overall water splitting.^69^ The aza‐CMP/C_2_N heterostructures were prepared through mixing and subsequently annealing of the CMP and C_2_N nanosheets. The as‐obtained stacked nanosheets of aza‐CMP/C_2_N with abundant overlapped areas can be revealed by transmission electron microscopy (TEM) images in **Figure** **9**a. X‐ray absorption near‐edge structure (XANES) spectroscopy, high‐angle annular dark‐field scanning transmission electron microscopy (HAADF‐STEM) image, and elemental mapping are applied to prove the interlayer interactions and uniform combination of aza‐CMP and C_2_N nanosheets (Figure 9b). The study on photocatalytic performance revealed that H_2_ and O_2_ (molar ratio: 2:1) are simultaneously produced from pure water with aza‐CMP/C_2_N heterostructures as photocatalysts under visible light (>420 nm) irradiation, while both aza‐CMP and C_2_N components are inactive. Furthermore, aza‐CMP/C_2_N heterostructures with the mass ratio of 1:1 exhibit the optimum photocatalytic performance (H_2_ production rate: 5.0 mmol h^−1^, solar‐to‐hydrogen conversion efficiency: 0.23%, apparent quantum efficiency at 600 nm: 4.3%) (Figure 9c). Figure 9d provides the energy band alignment of aza‐CMP/C_2_N heterostructures, which indicates that the photogenerated electrons in the conduction band of aza‐CMP are promptly recombined with the photogenerated holes at the valence band of C_2_N at their interface, whereas other photogenerated electrons and holes participated in the redox of water. Thanks to the as‐constructed heterostructures, both charge separation and transportation are facilitated in aza‐CMP/C_2_N composites (evidenced by the results of transient photocurrent and electrochemical impedance measurements) and thus enhanced the photocatalytic performance.

**Figure 10:**
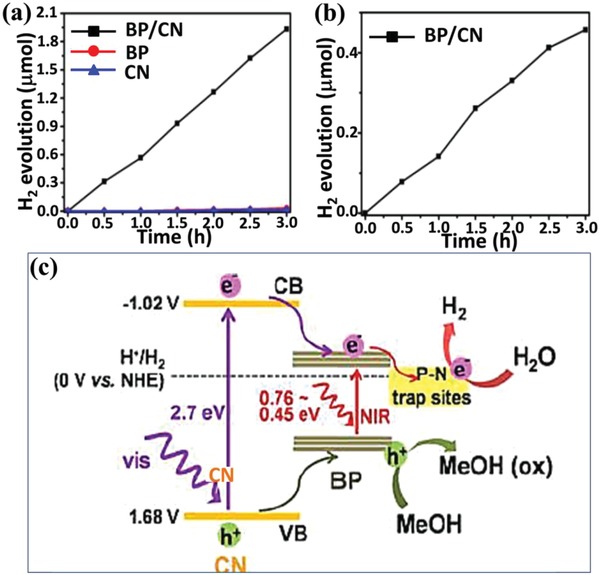
Photocatalytic H_2_ evolution based on different catalysts with a) >420 nm and b) >780 nm light irradiation; c) schematic diagram for photocatalytic H_2_ evolution using BP/CN. Reproduced with permission.^68^ Copyright 2017, American Chemical Society.

In the drive toward green chemistry, a series of metal‐free photocatalysts, such as g‐C_3_N_4_, black phosphorus (BP), boron nitride (BN), and boron carbide (BC), is highly preferred to be explored due to their high abundance, low cost, and excellent stability.^66^, ^67^, ^68^ So far, the exploration of efficient and stable metal‐free photocatalysts with broadband solar absorption for photocatalytic H_2_ production remains a big challenge. Zhu et al. successfully developed a 2D layered heterostructures (BP/CN) composed of black phosphorus and graphitic carbon nitride (CN) as metal‐free photocatalysts for H_2_ evolution in visible to near‐infrared (NIR) region for the first time.^68^ Compared with single component (BP or CN), BP/CN heterostructures exhibit significantly enhanced photocatalytic activity, H_2_ evolution reached 1.93 µmol under >420 nm light irradiation and 0.46 µmol under >780 nm light irradiation for 3 h (**Figure** **10**a,b). It is mainly attributed to the efficient charge transfer at the interface based on strong interface interaction between BP and CN, which inhibits the recombination and improves the separation of photogenerated e^−^–h^+^ (Figure 10c). Under visible light excitation, a charge transfer from CN to combined BP induced by heterojunction interfacial effect in BP/CN can be confirmed by the results of time‐resolved diffuse reflectance spectroscopic measurements. In the case of NIR excitation, only BP is excited and long lifetime is resulted from the efficient electron‐trapping by P−N coordinate bond at the heterojunction interface.

#### 4.1.2 Photocatalytic Pollutant Degradation

With the development of industry, mass discharge of poisonous wastes (such as dyes, antibiotics, and agrochemicals) has become a severe threat of water resource and human health worldwide.^77^ Besides improving the environmental laws and regulations, the development of ecofriendly solutions for eliminating pollution is desperately needed. Among the possible solutions, photocatalytic decomposition of organic contaminants through in situ generated highly reactive species is believed to be green, cost‐effective, and promising approach to address pollution problems.^78^ Although multiple types of single‐ingredient photocatalysts have been developed, most of them suffer from poor photocatalytic activity and are not efficient enough for practical application. A series of 2D/2D layered heterostructures has been developed for organics photodegradation, exhibiting promising application values.^79^, ^80^, ^81^, ^82^, ^83^, ^84^

**Figure 11:**
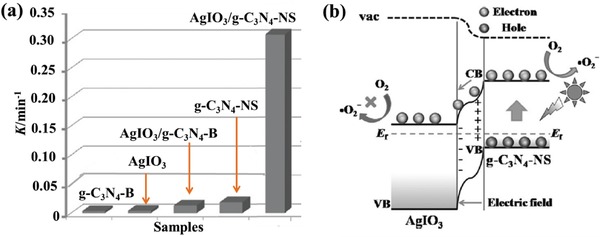
a) Photocatalytic degradation kinetics of RhB by the as‐prepared photocatalysts under visible‐light irradiation; b) schematic illustration of charge separation at the interface of AgIO_3_/g‐C_3_N_4_‐NS under visible‐light irradiation. Reproduced with permission.^81^ Copyright 2015, WILEY‐VCH.

2D/2D heterostructures composed of AgIO_3_ and g‐C_3_N_4_ nanosheets (AgIO_3_/g‐C_3_N_4_‐NS) were successfully synthesized for photocatalytic wastewater treatment under visible‐light irradiation.^81^ Graphite‐like carbon nitride nanosheets (g‐C_3_N_4_‐NS) as a polymeric organic semiconductor material exhibit good visible light response. The photocatalytic activity of AgIO_3_/g‐C_3_N_4_‐NS heterostructures is significantly higher than single AgIO_3_ or g‐C_3_N_4_ nanosheets toward the degradation of organic dyes. Notably, the reaction rate constant of rhodamine B (RhB) degradation over the as‐prepared AgIO_3_/g‐C_3_N_4_‐NS sample is almost 22.86 times higher than that of heterostructures composed of AgIO_3_ and bulk g‐C_3_N_4_ (AgIO_3_/g‐C_3_N_4_‐B), which indicates the importance of 2D dimensionality for the construction of heterojunction photocatalysts (**Figure** **11**a). A possible photocatalytic mechanism is also proposed in this work: the photoinduced electrons on the CB of g‐C_3_N_4_‐NS can migrate to the CB of AgIO_3_ under visible‐light irradiation, which promote the separation of photogenerated e^−^–h^+^ in g‐C_3_N_4_‐NS (Figure 11b). The significantly decreased peak intensity in steady‐state PL spectra and increased lifetime of charge carriers in the time‐resolved transient PL spectra of AgIO_3_/g‐C_3_N_4_‐NS compared with that of single C_3_N_4_‐NS, indicates the improved electron transport and charge‐separation efficiency at the heterojunction interface. Considering the reduction potential of O_2_/^•^O_2_^−^, the oxidative decomposition of dye should be attributed to the holes on the VB of g‐C_3_N_4_‐NS and ^•^O_2_^−^ generated by the reduction of O_2_ by the electrons on the CB of g‐C_3_N_4_‐NS. In another work, different amount of BiOCl nanoplates was applied to couple with C_3_N_4_ nanosheets through a simple calcination route. The face‐to‐face contact interface of the as‐prepared BiOCl/C_3_N_4_ heterostructures was characterized and the importance of contact area of the two components has been discussed in detail through comparative research.^82^ A much larger photocurrent intensity and weakened PL intensity of BiOCl/C_3_N_4_ heterostructures as compared with those of C_3_N_4_ nanosheets indicate the promoted separation and transfer of photogenerated electrons at the interface. BiOCl/C_3_N_4_ heterojunction photocatalysts with loading of 70% BiOCl exhibit the highest methyl orange photodegradation performance under visible‐light irradiation. In addition, Wang et al. fabricated g‐C_3_N_4_/Bi_2_WO_6_ 2D/2D heterostructures composed of ultrathin g‐C_3_N_4_ nanosheets and monolayer Bi_2_WO_6_ nanosheets for degradation of ibuprofen under visible light irradiation.^83^ This work reveals that highly active photodegradation system may be developed through steering the charge separation, transportation, and consumption at the atomic level.

Bera et al. developed a series of heterostructures composed of reduced graphene oxide (RGO) and CdS with different dimensionality (RGO/CdS) for the photocatalytic degradation of methyl blue (MB).^80^ Graphene as a typical 2D layered material with atomic thickness has excellent charge mobility, which can provide conductive electron channels for the separation of photogenerated charges in heterojunction photocatalyst composed of graphene and semiconductor.^79^, ^80^, ^84^ By using terephthalic acid (TA) as a probe, OH^•^ radicals are proved to be definitely produced active species for photocatalytic decomposition. Therefore, the enhanced activity of the RGO/CdS system compared with single CdS may be explained as following: under visible light irradiation, the photogenerated electron in the CB of CdS is transferred to the reduced graphene surface, and reacts with the adsorbed O_2_, which subsequently generates ^•^O_2_^−^ and OH^•^ radical. The oxidability of both OH^•^ and photogenerated hole contributes to the photocatalytic decomposition of MB dye molecules. The charge transfer from CdS to RGO happens at the interface of RGO/CdS heterostructures, as confirmed by the significantly quenched photoluminescence of CdS components, promoting the separation of photogenerated charges and thus enhancing the photocatalytic activity.

**Figure 12:**
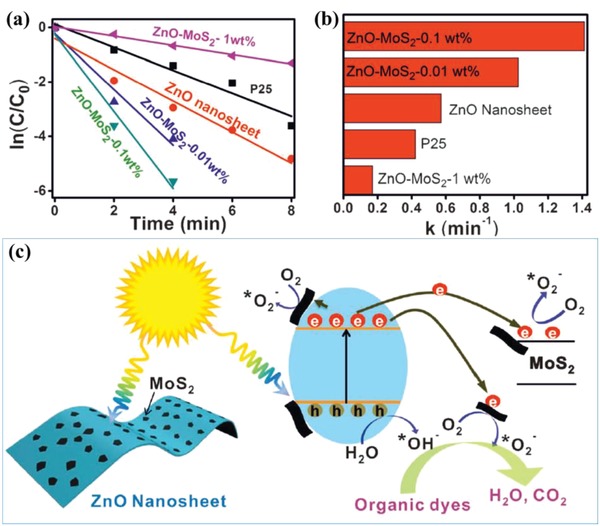
a) The ln (C/C_0_) versus time curves of MB with various photocatalysts: ZnO/MoS_2_ heterostructures with different loading amounts of MoS_2_ (mass ratio is 0, 0.01, 0.1, and 1 wt%) and commercial P25; b) the apparent rate constants of MB photodegradation with various photocatalysts; and c) schematic illustration of photocatalytic mechanisms of ZnO/MoS_2_ heterostructures. Reproduced with permission.^85^ Copyright 2014, WILEY‐VCH.

In some cases, the photocatalytic performance of pollutant degradation may be enhanced by a synergetic effect between heterojunctions and other structure engineering. Liu et al. developed a 2D/2D nanocomposite photocatalyst (ZnO/MoS_2_) based on P‐doped ZnO nanosheets with large surface area and 2D MoS_2_ for efficient photodegradation of organic dyes.^85^ ZnO/MoS_2_ heterostructures with different loading amounts of MoS_2_ (mass ratio is 0, 0.01, 0.1, and 1 wt%) and commercial P25 are used as photocatalysts for the photodegradation measurements, which are carried under the same experiment conditions (**Figure** **12**a,b). The comparison results reveal that ZnO/MoS_2_ with a small loading amount of MoS_2_ (0.1 wt%) would significantly enhance the photocatalytic activity compared with that of pure ZnO nanosheets. When the loading amount of MoS_2_ is increased from 0.01 to 0.1 wt%, the photocatalytic activity of ZnO/MoS_2_ is further increased, but decreased after the loading amount of MoS_2_ is further increased to 1 wt%. A large loading amount of MoS_2_ would block the sunlight, which is applied to drive the photodegradation of MB, and thus reduce the photocatalytic activity. A photocatalytic mechanism of ZnO/MoS_2_ heterostructures is proposed in Figure 12c. Driven by the interfacial effect between ZnO and MoS_2_, the photogenerated electrons would transfer from the CB of ZnO to the CB of MoS_2_, which significantly improve the separation of carriers and thus enhance the catalytic performance. The enhanced transfer and separation of photogenerated electrons and holes induced by interfacial effect can be confirmed by the increased photocurrent density of MoS_2_/ZnO heterostructures after the introduction of MoS_2_ components. The photogenerated charges react with O_2_ and H_2_O after transfer to the surface of catalyst, and the as‐produced highly reactive radicals (hydroxyl and superoxide anion radicals) further degrade the dye molecules. Besides the heterojunction effect, the phosphorous doping induced defects in ZnO nanosheets also promote the photocatalytic performance because they can improve the light absorption through the introduction of energy level between band gap.

### 4.2 Electrocatalysis

Electrocatalysis is a kind of efficient energy conversion technology through accelerating the electrochemical reaction on the electrode. It is believed to be promising for the development of advanced energy conversion system for solving the problems of energy shortage and environmental pollution and realizing the sustainable development of society. Water cycle and the carbon cycle are two main energy‐related applications in electrocatalysis and have been applied to various clean energy devices. For water cycle, oxygen reduction reaction (ORR) is the core reaction in fuel cells and metal−air batteries;^86^ HER and OER are the core reactions of water electrolysis cells that can produce ultrahigh‐purity hydrogen as clean fuel.^87^ For carbon cycle, reducing CO_2_ electrochemically and directing methanol/ethanol fuel cells are promising future energy systems.^88^

In general, advanced electrocatalyst with the features of good electrical conductivity, high activity, and long‐term stability is still urgently needed for practical applications.^89^ 2D nanomaterials are quite promising in electrocatalytic applications, due to not only their unique physical, chemical, and electronic properties, but also the dependence of electrocatalytic activity on the properties of catalysts' surface and interface.^24^, ^25^, ^39^ The design of 2D/2D heterojunctions has been proved to be a kind of effective interface engineering method for developing high‐performance electrocatalysts. The electronic states, chemical properties, and electrical conductivity could be significantly improved by constructing a highly coupled interface for better electrocatalytic performance.

**Figure 13:**
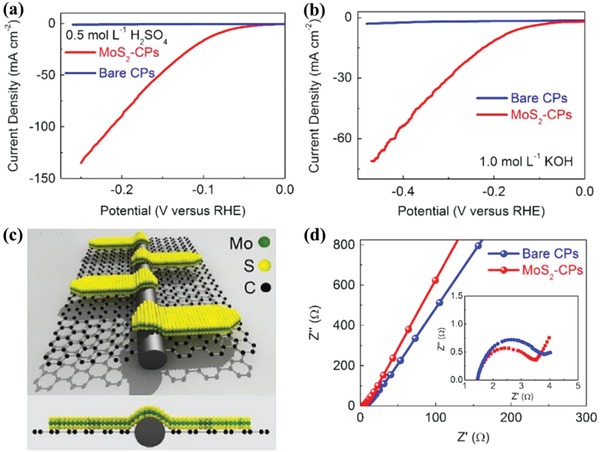
Polarization curves of MoS_2_‐CPs and bare CPs in N_2_‐saturated a) 0.5 mol L^−1^ H_2_SO_4_ and b) 1.0 mol L^−1^ KOH electrolyte with a sweep rate of 0.5 mV s^−1^; c) schematic illustration of MoS_2_‐CPs; and d) electrochemical impedance spectroscopy (EIS) spectra of MoS_2_‐CPs and bare CPs at low frequency with −0.1 V versus reversible hydrogen electrode (RHE) in N_2_‐saturated 0.5 mol L^−1^ H_2_SO_4_ electrolyte. Inset: corresponding EIS spectra at high frequency. Reproduced with permission.^16^ Copyright 2015, Elsevier.

Recently, Ye et al. successfully fabricated a kind of ultrathin MoS_2_ nanosheet decorated carbon paper (MoS_2_‐CP) through facile two‐step synthetic process: i) direct transformation of biomass bacterial cellulose films into nitrogen‐doped carbon papers, and ii) solution‐phase deposition of MoS_2_ nanosheets.^16^ Such highly coupled MoS_2_‐CPs heterostructures exhibit superb catalytic activity and stability when used as electrode for HER in both acid and basic media (**Figure** **13**a,b). During the catalytic process, the proton reduction kinetics should be mainly originated from the supported nanosheets in MoS_2_‐CPs electrode due to the fact that bare CPs are inactive at low potential (Figure 13a,b). In the as‐fabricated MoS_2_‐CPs heterostructures, MoS_2_ nanosheets are engineered as defect‐rich, small‐size, and ultrathin structure (Figure 13c), which ensure the abundance of exposed edges and defects with catalytic active sites for HER. Such success of structure engineering of MoS_2_ should be mainly attributed to the strong interaction at the interface between MoS_2_ and graphene in carbon papers. In addition, the hierarchical structure of CPs is beneficial for the mass transfer and hydrogen gas release, and its conductivity is not disturbed by the decorated MoS_2_ nanosheets (Figure 13d).

Tsai et al. investigated the effect of graphene support on the electrocatalytic HER of MoS_2_ in MoS_2_/graphene 2D/2D heterostructures through using periodic density functional theory, employing the new BEEF‐vdW functional.^90^ The long‐range van der Waals forces between MoS_2_ and graphene support were taken into account in this work. The authors found that the interaction (involving vdW forces) between MoS_2_ and graphene could significantly influence the hydrogen binding energy and thus enhancing the performance of electrocatalytic HER of MoS_2_/graphene. It was demonstrated that the electrocatalytic activity at the Mo‐edge of MoS_2_ would be determined by the interaction with graphene support, which can be controlled by modifying the electronic structures of graphene. The electronic structures of graphene would be varied with different defect density,^91^ element doping,^92^ or metal supporting in graphene.^93^

The development of bifunctional electrocatalysts with high activity for catalyzing both HER and OER is an important research topic for electrocatalytic water splitting, due to not only the requirement of further enhanced efficiency but also the high cost induced by different catalysts (such as different equipment and processes).^94^, ^95^, ^96^ A NiS/Ni_2_P 2D/2D heterostructure as bifunctional electrocatalyst for efficient electrocatalytic water splitting was successfully prepared through three‐step synthetic method composed of hydrothermal, sulfurization, and phosphorization process.^96^ Ni‐based sulfides and phosphides as earth‐abundant electrocatalysts for both HER and OER have been widely investigated in water‐splitting applications.^97^ Compared with single NiS or Ni_2_P, NiS/Ni_2_P heterostructures exhibited extremely higher catalytic performance (overpotentials: 111 and 265 mV, respectively, for the HER and OER; current density: 20 mA cm^−2^) under the same conditions. A shift of the X‐ray photoelectron spectroscopy (XPS) peaks assigned to Ni 2p3/2 and Ni 2p1/2 to lower binding energy after merging NiS with Ni_2_P components compared with those of NiS indicates the charge redistribution induced by strong electronic interactions between NiS and Ni_2_P, which would significantly influence the chemical properties and thus the catalytic activity. Strong electronic interactions, abundant active sites, and low interfacial resistance all merit the electrocatalytic activity of NiS/Ni_2_P heterostructures.

**Figure 14:**
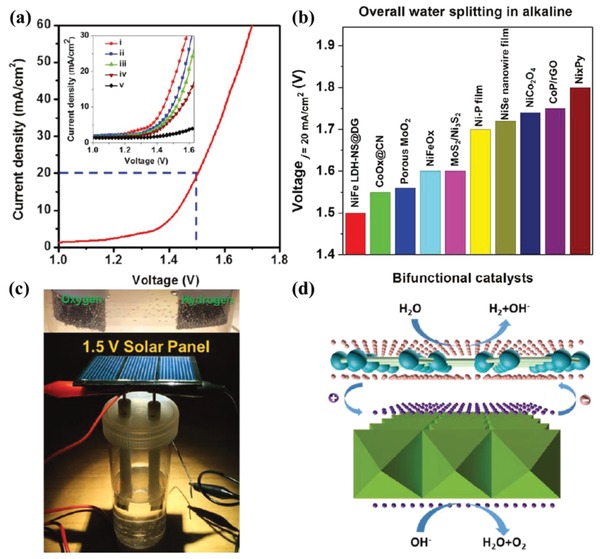
a) Polarization curves of Ni‐Fe LDH‐NS@DG as bifunctional catalyst for overall water splitting in 1 m KOH (loaded on Ni foam with 2 mg cm^−2^), inset: comparison of different catalysts (i: Ni‐Fe LDH‐NS@DG, 2 mg cm^−2^ loading; ii: Ni‐Fe LDH‐NS@DG with 1 mg cm^−2^ loading; iii: Ni‐Fe LDH‐NS@NG with 2 mg cm^−2^ loading; iv: Ni‐Fe LDH‐NS@G with 2 mg cm^−2^ loading; and v: bare Ni foam electrode); b) comparison of the required voltage (current density: 20 mA cm^−2^) of various noble metal free bifunctional electrocatalysts for overall water splitting; c) picture of a water‐splitting device assisted by solar; and d) schematic illustration of electrocatalytic mechanism of Ni‐Fe LDH‐NS@DG for overall water splitting based on the density functional theory (DFT) calculation results (pink sphere: electron, purple sphere: hole). Reproduced with permission.^95^ Copyright 2017, WILEY‐VCH.

Jia et al. developed a bifunctional 2D/2D composite electrocatalyst (Ni‐Fe LDH‐NS@DG) composed of Ni‐Fe layered double hydroxide nanosheets (Ni‐Fe LDH‐NS) and defective graphene (DG) with rich defects through electrostatic for efficient alkaline water splitting.^95^ As shown in **Figure** **14**a, as a bifunctional catalyst, the as‐prepared Ni‐Fe LDH‐NS@DG heterostructures exhibit significantly better electrocatalytic activity than that of Ni‐Fe LDH‐NS@NG (NG: nitrogen‐doped graphene) and Ni‐Fe LDH‐NS@G (G: pristine graphene), and higher loading (2 mg cm^−2^) of Ni‐Fe LDH‐NS@DG catalyst results in better performance. Compared with a series of state‐of‐the‐art non‐noble metal bifunctional electrocatalysts,^98^ Ni‐Fe LDH‐NS@DG heterostructures are proved to be the most efficient for overall alkaline water splitting at room temperature (Figure 14b). A water‐splitting device assisted by solar was also fabricated to evaluate the solar‐water electrolysis performance of LDH‐NS@DG, which could be clearly observed as driven by a 1.5 V solar panel (Figure 14c). Both of the defect sites in DG and charge transfer driven by interfacial effect contribute to the localized electrons' accumulation on DG and holes' accumulation on Ni‐Fe LDH‐NS, which is favorable to HER and OER, respectively (Figure 14d).

### 4.3 Organic Synthesis

Organic synthesis, especially the ones related to the production of chemicals closely linked to our life, is in urgent need of clean, safe, and environmentally benign reaction routes for sustainable development of human society, due to the pollution resulting from the traditional synthesis.^99^ Organic synthesis driven by light has been developed and attracts increasing attention due to its mild reaction conditions and high selectivity from the point of view of green chemistry.^100^ After the photoexcitation of semiconductor‐based catalysts, electron–hole pairs are produced and utilized for redox reactions in organic synthesis. During the reaction process, besides photogenerated electron/hole, various reactive species (such as hydroxyl, superoxide, and oxyhydoxyperoxide radicals) may be formed through capturing electron scavengers (such as oxygen) by photogenerated electrons for various reactions, such as selective reduction, oxidation, C—H activation, and C—C or N—N coupling reactions.^101^ Among the developed catalysts for light‐driven organic synthesis, 2D/2D heterostructures show great potential for enhancing reaction efficiency and developing valuable light‐driven organic transformation routes by using the photogenerated charge carriers at the interface as redox reagents or initiators.

Through immobilizing ZnIn_2_S_4_ nanosheets onto mesoporous P‐doped graphite carbon nitrogen (P‐C_3_N_4_) nanosheets, P‐C_3_N_4_/ZnIn_2_S_4_ 2D/2D heterostructures were rationally developed for the reduction of 4‐nitroaniline to the corresponding 4‐phenylenediamine under visible light irradiation.^102^ The ternary chalcogenide ZnIn_2_S_4_ with a narrow band is widely studied visible‐light responsive catalyst, and also exhibits stability toward light. P‐C_3_N_4_ nanosheets are proved to possess the characteristics of enhanced light harvesting, shorter diffusion distance of charge carrier pairs, and reduced interior defects due to the structure design of mesoporous nanosheets and phosphorus doping. The as‐prepared P‐C_3_N_4_/ZnIn_2_S_4_ heterostructures exhibit superior catalytic activity due to not only the above‐mentioned merits of two components, but also efficient charge transfer and separation‐induced heterojunction interfacial effect, which could be evidenced by the enhanced photocurrent response, smaller semicircle and lower the PL intensity of P‐C_3_N_4_/ZnIn_2_S_4_ compared with those of single P‐C_3_N_4_ or ZnIn_2_S_4_. In another work, CdS/g‐C_3_N_4_ 2D/2D heterostructures were developed for a coupled system of reducing nitrobenzene to aniline and selective oxidizing aromatic alcohols to aromatic aldehydes under visible light illumination.^103^ In such a coupled reaction system with both selective reduction and oxidation process, the photogenerated electrons and holes can not only perform their own role, but also realize the boosted separation and transfer efficiency, which are beneficial for their catalytic activity. In this work, the selective reduction and oxidation reactions were proved to be achieved by direct holes and electrons, respectively, rather than intermediate of active radicals. CdS/g‐C_3_N_4_ with 10 wt% CdS exhibits the optimal catalytic efficiency under visible light illumination.

**Figure 15:**
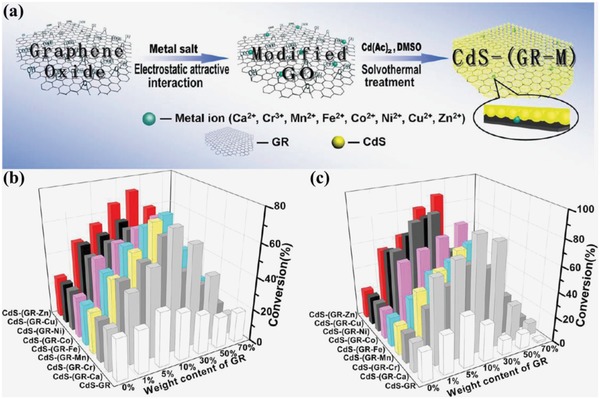
a) Schematic illustration of synthetic process of CdS‐(GR‐M) (M: metal ions of Cr^3+^, Mn^2+^, Ca^2+^, Ni^2+^, Cu^2+^, Fe^2+^, Co^2+^, and Zn^2+^); photocatalytic performance of CdS‐(GR‐M) (M: metal ions of Cr^3+^, Mn^2+^, Ca^2+^, Ni^2+^, Cu^2+^, Fe^2+^, Co^2+^, and Zn^2+^) with different weight addition ratio of GR, CdS‐GR, and CdS for b) photocatalytic oxidation of benzyl alcohol and c) reduction of 4‐nitroaniline under visible light irradiation (λ > 420 nm; 2 h and 80 min, respectively). Reproduced with permission.^104^ Copyright 2014, American Chemical Society.

Xu and co‐workers' research proved that the optimization of interfacial composition may significantly boost the catalytic performance for light‐driven organic synthesis through introducing some mediators (metal ions or noble metals) into the interfacial layer matrix between the two components in 2D/2D heterostructures.^104^, ^105^ They successfully boosted the catalysts' efficiency for aerobic selective oxidation of alcohol and anaerobic reduction of nitro compound under visible light irradiation through introducing a small amount of metal ions into the interfacial layer matrix between graphene and semiconductor in the 2D/2D graphene based heterostructures (CdS‐GR) with intimate interfacial contact.^104^ As a typical synthetic method, graphene oxide (GO) is prepared by Hummers' method first and then modified by proper amount of metal ions based on the oxygenated functional groups on the surface of GO with negative charges, which possess electrostatic attractive interaction with metal ions (Cr^3+^, Mn^2+^, Ca^2+^, Ni^2+^, Cu^2+^, Fe^2+^, Co^2+^, and Zn^2+^) (**Figure** **15**a). The as‐prepared modified heterostructures are named CdS‐(GR‐M) (M: metal ions of Cr^3+^, Mn^2+^, Ca^2+^, Ni^2+^, Cu^2+^, Fe^2+^, Co^2+^, and Zn^2+^). Two different probe reactions of aerobic oxidation of alcohol and anaerobic reduction of nitro compound are applied to compare the photocatalytic activity of CdS‐(GR‐M) and CdS‐GR under visible light irradiation (Figure 15b,c). Similar research results concluded that the photocatalytic activity is improved moderately after coupling GR compared with single CdS, and improved significantly after modifying a few amounts of metal ions (including Cr^3+^, Mn^2+^, Ca^2+^, Ni^2+^, Cu^2+^, Fe^2+^, Co^2+^, and Zn^2+^) into the interface between GR and CdS. Furthermore, the weight addition ratio of GR is increased from ≤5% to 30% after the modification of metal ions, which is a breakthrough of the limitation of shielding effect in GR‐based composite photocatalysts. The enhanced catalytic activity should be mainly attributed to the optimization of heterojunction interfacial effect induced by introducing metal ions as “mediator” for optimized transfer pathway, improved transfer efficiency, and prolonged lifetime of photogenerated charge across the interface. Furthermore, the introduced metal ions can partially offset the “shielding effect” of GR, which is a weakened light absorption due to its opacity resulting from high weight addition.^106^ In another work from the same group, they significantly enhanced the catalytic activity of the 2D/2D heterostructures composed of graphene and CdS (graphene/CdS) for both aerobic selective oxidation of alcohols and anaerobic reduction of nitro compounds under visible light irradiation through inducing Pd nanoparticles as mediator into the interlayer matrix of graphene/CdS.^105^ The twofold effects of the introduced Pd are demonstrated: i) Pd nanoparticles as electron reservoir directly trap electrons photoexcited from CdS, and ii) Pd nanoparticles promote the electron relay in catalysts.

**Figure 16:**
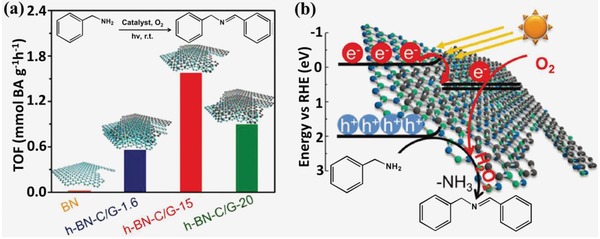
a) TOF values of h‐BNC/G‐*x* (*x* = 1.6, 15, 20; weight percentage of glucose in reactants) and pristine BN; b) schematic illustration of reaction mechanism over the h‐BN‐C/G. Reproduced with permission.^21^ Copyright 2018, WILEY‐VCH.

Due to the advantages of low cost and excellent stability, metal‐free 2D/2D heterostructures also have been developed for catalyzing light‐driven organic synthesis.^9^, ^21^ Zhang et al. recently developed a metal‐free and thinnest (atomic‐scale) heterostructures (h‐BN‐C/G) composed of BN and graphene for catalyzing the highly selective and oxidative coupling reactions of amines under visible light irradiation.^21^ The key step of the coupling amines reaction is to activate the molecular oxygen to assist the following dehydrogenation steps. Mild reaction condition under room temperature was successfully realized in this work although high temperature is usually needed in this step due to the energy barrier. It was demonstrated that the efficient reactivity of the as‐prepared composite catalysts should be mainly attributed to the promoted transfer and separation of the photogenerated charges for directly activating the molecular oxygen and substrate due to the heterojunction effect as evidenced by the results of photoluminescence and transient photovoltage. A series of h‐BN‐C/G‐*x* heterostructures with different carbon contents were prepared for comparison by changing the weight percentage of glucose in the mixture of reactants (*x* = 0, 1.6, 15, 20; weight percentage of glucose in reactants) with fixed amounts of urea. h‐BN‐C/G‐15 was proved to be the optimized sample and offered the highest turnover frequency (TOF) value (1.58 mmol BA g^−1^ h^−1^) for the coupling reactions of amines to imines under the same reaction conditions as shown in **Figure** **16**a. To confirm the reaction mechanism over h‐BN‐C/G, scavenger butylated hydroxytoluene (superoxide radical scavenger), mannitol (hydroxyl radical scavenger), catalase (hydrogen peroxide scavenger), and carotene (singlet oxygen) were employed to conform the active species for activating oxygen gas. As a result, hydrogen peroxide was proved to be real active intermediate for activating oxygen gas to hydrogen peroxide for further oxidative coupling of benzylamine into imine under visible light irradiation (Figure 16b).

## 5 Conclusions and Perspectives

In summary, this review highlights the general strategies and recent progress in the 2D/2D heterojunctions and heterostructures, which are excellent candidates for fundamental research and potential catalytic applications due to their unique structural physicochemical and electronic properties: i) integrating the merits of their components, such as 2D ultrathin structure, large surface area, and physicochemical/electronic properties; ii) the separation or transfer of charges can be promoted for desirable properties or functions; and iii) versatile options (including component, thickness, defect, fabrication technology, contact distance, etc.) can be designed to tune properties and thus impact application performance. Driven by the above‐mentioned advantages and potential values of 2D/2D heterostructures, increasing amounts of remarkable achievements have been made in the past few years. Nevertheless, in light of the challenges remaining in practical applications of catalysis, e.g., catalytic efficiency, selectivity associated with yields and pollution, environmentally friendly, and cost, there is still much work to pursue with the aims of developing desired catalyst and reaction system for practical applications.

Catalysts hold the key for boosting the efficiency of catalytic reaction process, which is the threshold of commercially feasible applications needed to overcome. 2D/2D heterostructures as photocatalysts have to be designed with the aim to promote the light harvesting, separation/transfer of charge carrier, redox reactivity, etc. Besides adequately taking advantage of 2D components through the fine tuning of the structural, compositional, band gap and surface reaction sites, interfacial engineering at nanoscale of 2D/2D heterostructures is expected to further facilitate the photocatalytic performance. Although many opportunities exist for 2D/2D heterostructures as electrocatalysts, this field is also faced with many challenges and research space: i) synthetic methods are further needed to be optimized to satisfy the industrial and commercial requirements; ii) characterization techniques should be more advanced for the structural, physicochemical properties and performance for such ultrathin heterostructures; and iii) clearer understanding of the work mechanism, especially the reaction intermediates, of 2D/2D heterojunction electrocatalysts is still desired. Besides the above‐mentioned optimization scheme of catalyst design, high product yield/selectivity and the development of new organic synthesis system are all the keys in further research.

Thanks to the rapid advances and rich knowledge accumulated in 2D materials and heterostructures in the past decade, one may expect that 2D/2D heterostructures would play an important role in solving energy and environmental challenges.

## Conflict of Interest

The authors declare no conflict of interest.
